# Synthesis of 1,2-phenylenediamine capturing molecule for the detection of diacetyl

**DOI:** 10.1016/j.dib.2017.09.072

**Published:** 2017-10-06

**Authors:** Lucia Marri, Anita M. Jansson, Caspar E. Christensen, Ole Hindsgaul

**Affiliations:** Carlsberg Research Laboratory, J.C. Jacobsens Gade 4, DK-1799 Copenhagen V, Denmark

## Abstract

Here we describe the design of 1,2-phenylenediamine capturing molecule and the synthesis steps necessary for its preparation. The designed 1,2-phenylenediamine derivative is able to capture diacetyl in solution, as shown by ESIMS, forming a chemical adduct, 1-4-quinoxaline. The methyl esters of diacetyl-adduct (DAA) and pentanedione-adduct (PDA) are incorporated to the lysines in BSA and the conjugate used for antibody screening and selection. In the research article is described an enzyme-linked immunosorbent assay developed to detect and quantify diacetyl in complex media.

**Specifications Table**

**Table:** 

Subject area	*Chemistry*
More specific subject area	*Chemical synthesis, assay development*
Type of data	*Text file, chemical structure*
How data was acquired	ESIMS was recorded on a Bruker Esquire 3000-Plus Ion Trap instrument.
	MALDI-TOF-MS was recorded on Bruker Microflex instrument.
	HRESIMS was recorded on a Q-Tof Ultima instrument from Micromass.
	^1^H spectra were recorded at 20 °C on a Bruker 400 MHz instrument.
Data format	*Raw and analyzed data*
Data accessibility	*Data is with this article.*
Related research article	L. Marri, A. M. Jansson, C. E. Christensen, O. Hindsgaul, *Analytical Biochemistry,* in press.

**Value of the Data**

• The data explains the design of 1,2-phenylenediamine capturing molecule and could be used by others researchers.
• All the steps for the synthesis of the compounds here described and the methods can be followed by other researchers.
• The methods for incorporation of the diacetyl-adduct (DAA) and pentanedione-adduct (PDA) to the lysines in BSA can be used for preparation of other BSA-conjugates.

## 1 Data

The synthesis steps necessary for the preparation of the 1,2-phenylenediamine derivative (linker diamine) are described in details. The synthesis of the methyl esters of diacetyl-adduct (DAA) and pentanedione-adduct (PDA) and their incorporation in BSA to form the corresponding BSA-conjugates (BSA-DAA or BSA-PDA) are described.

## 2 Experimental design, materials and methods

### 2.1 General methods

Organic solvents were concentrated under reduced pressure at < 40 °C. Vacuum liquid chromatography was performed on silica gel (60 H, dried in oven at 120 °C) Reversed phase chromatography was performed on Sep-Pak Plus C_18_ cartridge (Waters, washed with 10 mL MeCN, 10 mL 50% MeCN_aq_ and 20 mL H_2_O before use). ESIMS was recorded on a Bruker Esquire 3000-Plus Ion Trap instrument with samples injected as solutions in MeCN-H_2_O 1:1 mixture. MALDI-TOF-MS was recorded on Bruker Microflex instrument, with sinapinic acid (10 mg/mL) in 30% MeCN in H_2_O containing 1% TFA, as matrix. HRESIMS was recorded on a Q-Tof Ultima instrument from Micromass. ^1^H spectra were recorded at 20 °C on a Bruker 400 MHz instrument using CDCl_3_ (7.27 ppm), CD_3_OD (3.31 ppm), CD_3_CN(1.94 ppm), or D_2_O (4.79 ppm) as internal standards.



4-Piperidone hydrate hydrochloride (2.8 g, 0.018 mmol) and pyridine (8.5 g, 8.7 mL, 0.108 mmol) was dissolved in dichloromethane (100 mL) with help of sonication. A solution of glutaric acid monomethylester chloride (6.0 g, 0.036 mmol) in dichloromethane (20 mL) was slowly added, and the resulting solution was stirred for 20 h at 20 °C. The reaction mixture was extracted with 2 M HCl (1 × 100 mL), saturated NaHCO_3_ (1 × 100 mL), brine (1 × 100 mL) and H_2_O (1 × 100 mL), followed by drying over NaSO_4_. Filtration, washing with dichloromethane and concentration gave **1** [1] (3.2 g, 78%, yellow oil).

^1^H NMR (400 MHz, CDCl_3_, δ ppm) 2.00 (dt, 2H), 2.43–2.51 (m, 8H), 3.68 (s, 3H), 3.77 (t, 2H, J = 6 Hz), 3.88 (t, 2H, J = 6 Hz). ESIMS calculated for C_11_H_17_NO_4_ 227.1, found: 228.2 [M+H]^+^.



A mixture of **1** (0.45 g, 2.0 mmol), trimethyl ortoformate (0.24 g, 0.25 mL, 2.3 mmol), methanol (14 mL) and *p*-toluenesulfonic acid (4 mg) was gently heated while the methyl formate formed was distilled off through a short vigreux column. When no more methyl formate was distilled off, the reaction mixture was cooled, made basic with a few drops of 2N sodium methoxide and was partitioned between ethyl acetate (50 mL) and water (50 mL). The organic phase was washed with brine (50 mL) and dried over potassium carbonate. Filtration, washing with ethyl acetate and concentration afforded the dimethyl acetate **2** (0.45 g, 83%) [2]. The crude product was used in the following step without further purification. ^1^H NMR (400 MHz, CDCl_3_, δ ppm) 1.63–1.69 (m, 2H), 1.84–1.92 (dt, 2H, J = 7 Hz), 2.29–2.35 (m, 4H), 3.13 (s, 6H), 3.35–3.40 (m, 2H), 3.50–3.55 (m, 2H), 3.60 (s, 3H). ESIMS calculated for C_13_H_23_NO_5_ 273.2, found: 274.0 [M+H]^+^.



A mixture of the dimethyl acetal **2** (150 mg, 0.55 mmol), 1,2-dihydroxybenzene (72 mg, 0.65 mmol) and toluene (8 mL) was brought to reflux and part of the solvent (2 mL) was distilled through a short vigreux column. The temperature was lowered to 60 °C and *p*-toluenesulphonic acid (3 mg) was added. Distillation of the solvent was slowly continued until pure toluene was being collected. During this operation, addition of more toluene (8 mL) was necessary to prevent that the reaction mixture become dry. Triethylamine (0.1 mL) was added to the cooled reaction mixture, which was then partitioned between ethyl acetate and water. The organic phase was washed with water (1 × 20 mL), 0.1 M sodium hydroxide (2 × 20 mL), brine (2 × 20 mL) and dried over potassium carbonate. Filtration, washing with ethyl acetate and concentration gave **3** (120 mg, 69%) [2]. The crude product was used in the following step without further purification. ^1^H NMR (400 MHz, CDCl_3_, δ ppm) 1.88–1.97 (m, 6H), 2.32–2.44 (m, 4H), 3.57–3.62 (m, 2H), 3.61 (s, 3H), 3.72–3.77 (m, 2H). ESIMS calculated for C_17_H_21_NO_5_ 319.1, found: 320.2 [M+H]^+^.



A solution of **3** (200 mg, 0.63 mmol) in acetic acid (1.5 mL) was added drop-wise under stirring into fuming nitric acid (2 mL) on an ice bath followed by stirring at room temperature for 1 h. The reaction mixture was then poured into ice-water (5 mL) and the formed precipitate was isolated by centrifugation. The precipitate was washed with water (2 × 2 mL) and dried by freeze drying. This gave **4** (190 mg, 74%) [3]. The crude product was used in the following step without further purification. ^1^H NMR (400 MHz, CDCl_3_, δ ppm) 1.89–1.98 (m, 2H), 2.01–2.10 (m, 4H), 2.36–2.44, (m, 4H), 3.61 (s, 3H), 7.23 (s, 2H). HRESIMS calculated for C_17_H_20_N_3_O_9_ 410.1200 [M+H]^+^, found: 410.1188.



A mixture of di-nitro compound **4** (81 mg, 0.20 mmol), stannous chloride di-hydrate (226 mg, 1 mmol) and 12 M HCl (0.12 mL) in ethyl acetate (12 mL) was heated at 50 ^o^C for 2 h. When adding additional 12 M HCl (0.12 mL) and ethyl acetate (20 mL) the product solidified and was isolated by centrifugation. The solid was washed with ethyl acetate (2 × 10 mL) and dried. This gave **5** (65 mg, 78%, yellow solid) [4], [5]. The crude product was used in the following step without further purification. ^1^H NMR (400 MHz, CD_3_OD, δ ppm), 1.77–1.86 (m, 2H), 1.86–1.91 (m, 2H), 1.94–1.99 (m, 2H), 2.32 (t, 2H, J = 7.2), 2.41 (t, 2H, J = 7.4) 3.60–3.70 (m, 4H), 6.58, (s, 2H). ESIMS calculated for C_17_H_23_N_3_O_5_ 349.2, found: 350.0 [M+H]^+^.



Compound **5** (10 mg, 0.024 mmol) and diacetyl (20 mg, 0.23 mol) was dissolved in water-acetic acid 2:1 (1 mL). The reaction mixture was stirred at room temperature for 1 hour. Dilution with 4 mL water followed by purification on SepPak (20–30% aq. CH_3_CN) gave the diacetyl adduct **6** (9 mg, 92%) [6]. ^1^H NMR (400 MHz, CDCl_3_, δ ppm) 1.74–1.81 (m,2H), 1.93–1.97 (m,2H), 2.00–2.04 (m,2H), 2.26–2.36 (m,4H), 2.51 (s,3H), 3.55 (s,3H), 3.57–3.61 (m,2H), 3.66–3.70 (m,2H), 7.10 (s,2H). ESIMS calculated for C_21_H_25_N_3_O_5_ 399.2, found: 400.1 [M+H]^+^.



Compound **6** (9 mg, 0.022 mmol) was dissolved in a minimal volume of dioxane (0.2 mL). Water was added (0.3 mL) followed by 2 M sodium hydroxide (0.3 mL) and the reaction mixture was stirred at room temperature for 15 min. The pH was adjusted to 4.2 with 1 M and 0.1 M HCl. Purification was then directly performed by reversed phase chromatography on a C_18_ SepPak cartridge. After washing with water the product was eluted with water-acetonitrile 1:1. Evaporation of the solvents gave **7** (8 mg, 95%). ^1^H NMR (400 MHz, CD_3_OD, δ ppm) 1.81–1.97 (m, 2H), 2.08–2.12 (m, 2H), 2.15–2.19 (m, 2H), 2.38 (t, 2H, 7.2 Hz), 2.54 (t, 2H, 7.4 Hz), 2.65 (s, 6H), 3.81–3.87 (m, 4H). HRESIMS calculated for C_20_H_23_N_3_O_5_ [M+H]^+^ 386.1716, found: 386.1704.



N,N´-disuccinimidyl carbonate (1.9 mg,7.4 μmol) dissolved in dry DMF (0.1 mL) and 4-(dimethy1amino)pyridine (1.5 mg, 12 μmol) dissolved in dry DMF (0.1 mL) were added to a solution of compound **7** (2 mg, 5.2 μmol) in dry DMF (0.4 mL) under magnetic stirring. Activation proceeded for 40 min. Dilution of reaction mixture with 0.01 M HCl to less than 10% DMF was followed by purification by reversed phase chromatography on a C_18_ SepPak cartridge. After washing with water-acetonitrile 9:1 and 8:1 the product was eluted with water-acetonitrile 1:1. Evaporation of the solvents gave **8** (2.2 mg, 88%) [7]. ^1^H NMR (400 MHz, CDCl_3_, δ ppm) 1.86–1.96 (m, 2H), 2.44 (t, 2H, 8 Hz), 2.51 (s, 6H), 2.63 (t, 2H, 7.5 Hz), 2.63 (s, 4H), 3.57–3.61 (m, 2H), 3.67–3.71 (m, 2H), 7.10 (s, 2H). ESIMS calculated for C_24_H_26_N_4_O_7_ 482.2, found: 483.1 [M+H]^+^.



Bovine serum albumin (2 mg, 0.03 μmol) was dissolved in 0.1 M phosphate buffer pH 7.5 and added to a solution of compound **8** (0.9 mg, 2 μmol) in DMSO (100 μL) and 0.1 M phosphate buffer pH 7.5 (3 mL). The reaction mixture was gently vortexed overnight at room temperature and purified by concentration using a Millipore MW CO 30 000 filter (4000 rpm/6 min).The protein on the filter was washed with water (4 × 4 mL) and lyophilisation gave **9** [6]. MALDI-TOF-MS found m/z 70 530, that corresponds to an incorporation of ~11 adducts per BSA.



Compound **5** (35 mg, 0.083 mmol) and pentanedione (35 g, μL) was dissolved in water-acetic acid 2:1 (2.5 mL). The reaction mixture was stirred at room temperature for 1 hour, and purification was then directly performed by reversed phase chromatography on a C_18_ SepPak cartridge. After washing with water, water-acetonitrile 9:1 and 8:2, the product was eluted with water-acetonitrile 1:1. Evaporation of the solvents gave **10** (27 mg, 79%) [6]. ^1^H NMR (400 MHz, CDCl_3_, δ.ppm) 1.28 (t, 3H, 7.5 Hz), 1.95–2.07 (m, 6H), 2.30–2.47 (m, 4H), 2.65 (s, 3H), 2.90 (q, 2H, 7.5) 3.60 (s, 3H) 3.76–3.82 (m, 2H), 3.76–3.82 (m, 2H) 7.23 (s, 1H), 7.27 (s, 1H). ESIMS calculated for C_22_H_27_N_3_O_5_ 413.2, found: 414.1 [M+H]^+^.



Compound **10** (7 mg, 0.017 mmol) was dissolved in a minimal volume of dioxane (0.2 mL). Water was added (0.3 mL) followed by 2 M sodium hydroxide (0.3 mL) and the reaction mixture was stirred at room temperature for 15 min. The pH was adjusted to 5 with 1 M and 0.1 M HCl. Purification was then directly performed by reversed phase chromatography on a C_18_ SepPak cartridge. After washing with water the product was eluted with water-acetonitrile 1:1. Evaporation of the solvents gave **11** (6.5 mg, 95%). ^1^H NMR (400 MHz, CDCl_3_, δ ppm) 1.26 (t, 3H, 7 Hz), 1.78–1.86 (m, 2H), 1.98–2.02 (m, 2H), 2.07–2.11 (m, 2H), 2.16 (t, 2H, 6 Hz), 2.59 (s, 3H), 2.89 (q, 2H, 7 Hz), 3.73–3.77 (m, 4H), 7.10 (s, 1H), 7.15 (s, 1H). ^1^H NMR (800 MHz, CDCl_3_, δ.ppm) 1.37 (t, 3H), 1.93, 2.11, 2.19 (m, 6H), 2.26, 2.53 (m, 4H), 2.69 (2, 3H), 2.99 (q, 2H) 3.70 (s, 3H) 3.86 (m, 4H), 7.20 (s, 1H), 7.25 (s, 1H). HRESIMS calculated for C_21_H_25_N_3_O_5_ [M+H]^+^ 400.1873, found: 400.1848.



N,N´-disuccinimidyl carbonate (3.3 mg, 13 μmol) dissolved in dry DMF (0.1 mL) and 4-(dimethy1amino)pyridine (3.3 mg, 27 μmol) dissolved in dry DMF (0.1 mL) were added to a solution of compound **11** (3.25 mg, 8 μmol) in dry DMF (0.1 mL) under magnetic stirring. Activation proceeded for 40 min. Dilution of reaction mixture with 0.01 M HCl to less than 10% DMF was followed by purification by reversed phase chromatography on a C_18_ SepPak cartridge. After washing with water-acetonitrile 9:1 and 8:1 the product was eluted with water-acetonitrile 1:1. Evaporation of the solvents gave **12** (3.5 mg, 87%) [7]. ^1^H NMR (400 MHz, CDCl_3_, δ ppm) 1.22 (t, 3H, 8 Hz), 1.85–1.95 (m, 2H), 1.91–2.02 (m, 4H), 2.43 (t, 2H, 8 Hz), 2.52 (s, 3H), 2.62 (m, 2H, 8 Hz), 2.67 (s, 4H), 2.84 (q, 2H, 8 Hz), 3.56–3.61 (m, 2H), 3.66–3.71 (m, 2H), 7.09 (s, 1H), 7.10 (s, 1H). ESIMS calculated for C_25_H_28_N_4_O_7_ 496.2, found: 497.1 [M+H]^+^.



Bovine serum albumin (3.8 mg, x μmol) was dissolved in 0.1 M phosphate buffer pH 7.5 (1.6 mL) and added to a solution of compound **12** (3.5 mg, 7 μmol) in DMSO (200 μL) and 0.1 M phosphate buffer pH 7.5 (0.8 mL). The reaction mixture was gently “shaken” overnight and purified by concentration using a Millipore MW CO 30 000 filter (4000 rpm/6 min).The residual protein on the filter was washed with water (4 × 4 mL) and lyophilisation gave **13** [6]. MALDI-TOF-MS found m/z 70 380, that corresponds to an incorporation of ~11 adducts per BSA.



To a mixture of **5** (7 mg, 17 µmol) in dioxane (300 uL) and water (100 uL), under argon, was added triethylamine (11,5 µl, 83 µmol) followed by di-*t*-butyl dicarbonate (11 mg, 50 µmol). The reaction mixture was stirred at room temperature for 3 h. The solvents were evaporated and the residue, dissolved in a minimal volume EtOAc, was purified by VLC (silica 60H). The product was eluated with 60–70% EtOAc on hexane. This gave **14** (7 mg, 67%). ^1^H NMR (400 MHz, CD_3_CN ppm) 1.40 (s, 18 H), 1.80–1.90 (m, 2H), 1.87–2.05 (m, 4H), 2.30–2.45 (m, 4H), 3.58–3.74 (m, 7H), 6.98 (s, 2H). ESIMS calculated for C_27_H_28_N_4_O_7_ 549.3, found: 550.1 [M+H]^+^, 572.1 [M+Na]^+^, 588.1 [M+K]^+^ (**Fig. 3**) [8].



Compound **14** (0,1 mg, 0.2 μmol) was stirred with 10% CH_2_Cl_2_ in TFA (70 uL) under argon for 10 min. Solvents were evaporated under a stream of argon followed by 5 min in speed-vac. The TFA-salt of the linker phenylenediamine, **15**, was directly used in capture reactions. ESIMS calculated for C_17_H_23_N_3_O_5_ 349.2, found: 350.2[M+H]^+^, 372.1[M+Na]^+^, 721.3[2M+Na]^+^ (**Fig. 3**) [8].

Reaction of compound **15** with diacetyl to afford diacetyl adduct **6**, analysed with ESIMS.

To a solution of compound **15** (~ 0.2 μmol) in water (20 μL) was added 10 μL of a solution of diacetyl in water (0.5 mg/100 μL). ESIMS after 2 min (dilution × 10 with MeCN-H_2_O 1:1) shows the diacetyl adduct product. ESIMS calculated for C_21_H_25_N_3_O_5_ 399.2, found: 400.2 [M+H]^+^ (**Fig. 3**) [8].
