# Supplementary material for: Synthesis of 1,2-phenylenediamine capturing molecule for the detection of diacetyl
Source: Data Brief. 2017 Oct 6;15:483–90. doi: 10.1016/j.dib.2017.09.072 (PMC5647469; doi:10.1016/j.dib.2017.09.072)
Supplement: Supplementary file 1 — Supplementary material [file mmc1.docx]

Lucia Marri

Carlsberg Research Laboratory

Gamle Carlsberg Vej 10

DK-1799 Copenhagen V

Denmark

+45 33275263

[Lucia.Marri@carlsberglab.dk](mailto:Lucia.Marri@carlsberglab.dk)

William Jakoby

Editor in Chief

Analytical Biochemistry: Methods in the Biological Sciences

July 27, 2017

Dear Prof. Jakoby,

Regarding the Data in Brief manuscript “Synthesis of 1,2-phenylenediamine capturing molecule for the detection of diacetyl” by Lucia Marri, Anita M Jansson, Caspar E Christensen and Ole Hindsgaul (Manuscript No.: DIB-D-17-00667), we declare that

- Our manuscript has not been published and is not under consideration for publication elsewhere.
- There are no conflicts of interest associated to this publication.

Sincerely,

Lucia Marri, PhD

Carlsberg Research Laboratory
